# Standardized Plant Extract Alleviates the Negative Effects of FMD Vaccination on Animal Performance

**DOI:** 10.3390/ani10030455

**Published:** 2020-03-09

**Authors:** Santi Devi Upadhaya, Yong Min Kim, Huan Shi, Josselin Le Cour Grandmaison, Alexandra Blanchard, In Ho Kim

**Affiliations:** 1 Department of Animal Resource and Science, Dankook University, No. 29 Anseodong, Cheonan, Choongnam 330-714, Korea; santi.upadhaya@gmail.com (S.D.U.); dydals3561@naver.com (Y.M.K.); sh30988@hotmail.com (H.S.); 2 Pancosma, A-One Business Center, La piece 3, CH-1180 Rolle, Switzerland; j.lecourgrandmaison@gmail.com (J.L.C.G.); Alexandra.Blanchard@pancosma.com (A.B.)

**Keywords:** FMD, immune, plant extract, performance, vaccination

## Abstract

**Simple Summary:**

Foot and Mouth Disease (FMD) is among the viral diseases causing poor growth performance and reduced immune status, leading to heavy economic losses in livestock. The vaccination of animals against FMD may lead to vaccination stress, thereby reducing the growth performance of animals. The growth promoting effects of a plant extract (consisting of capsicum and turmeric oleoresins) against FMD vaccinated growing pigs are evaluated in the present study. It was determined that the supplementation of the plant extract significantly improved performance and increased the antibody titer against FMD antigens. However, the immune parameters measured at days 10, 15, 20 and 25 post-FMD vaccination remained unaffected.

**Abstract:**

The present study was conducted to assess the efficacy of a plant extract (PE) on growth performance and immune status in foot and mouth disease (FMD)-vaccinated growing pigs. A total of 120 crossed ((Landrace × Yorkshire) × Duroc) growing pigs with an average initial body weight (BW) of 24.66 ± 2.34 kg and an average age of 70 days were randomized into three groups (10 pens; 4 pigs per pen per treatment) as follows: a nonvaccinated negative control group (NV), a FMD vaccinated group (OV), and a third group received a 0.0125% PE supplement after vaccination (PV), in a 6-week trial. The PV group receiving PE supplementation increased (*p* < 0.05) the BW compared with the OV group, and average daily gain (ADG) during days 1–14, overall and gain-to-feed ratio (G: F) in days 1–14, and dry matter (DM) digestibility at week 6 were higher (*p* < 0.05) in the PV compared with the OV group. A significant increase (*p* < 0.05) in haptoglobin concentration was observed in the OV group compared with the NV group at 25 days postvaccination. The inhibition percentage of antibodies against FMD in the sera reached above 50% in the PV group 5 days earlier than in the OV group. The findings suggest that the inclusion of PE in the diet promoted the performance of vaccinated growing pigs.

## 1. Introduction

Foot-and-mouth disease (FMD), caused by an RNA virus belonging to the Picornaviridae family, is a prevalent acute infectious disease affecting 77% of the global livestock population [1]. There are seven serologically and genetically distinguishable types of the virus, (O, A, C, Asia1, SAT1, SAT2, and SAT3); moreover, within each serotype, a large number of subtypes have evolved [2]. The FMD virus (FMDV) infects pigs, cattle, sheep, and goats which have cloven-hoofs, with devastating effects on the livestock industry [3]. FMD causes high morbidity and low mortality in adult animals, although high mortality also occurs in young stocks [4,5]. In the endemic region alone, production losses and vaccination costs were estimated to be between US $6.5 and 21 billion [1]. Therefore, as a preventive measure, FMD vaccinations are given to animals. Early exposure of an animal to a disease-causing organism allows the animal’s immune system to remember it, making the animal better able to quickly fight off infection when it comes into contact with the disease again. However, many types of adverse responses have been reported following vaccination, ranging from mild reactions to more serious side effects which can eventually affect the growth performance and production of animals. For instance, cases of postvaccination allergic reactions, edema, and vesicles in the teat were reported in a dairy cattle herd 8 days after the annual FMD vaccination, leading to losses in production and, therefore, economic loss for the producer [6]. The financial losses originating from the adverse effects of vaccination have been a serious concern for both farmers and primary prevention personnel [7]. Various causative factors have been reported to be associated with the adverse effects of the FMD vaccine, including the tolerance capacity of livestock, inappropriate vaccine production, transportation and storage, and improper practices [7]. In recent years, herbal immune-modulators have been considered safe alternatives that are totally free from animal or human pathogens, and have also been reported to show some beneficial effects in terms of cost, distribution, and production [8,9]. Thus, PE can be administered to animals to overcome the adverse effects of vaccinations while still eliciting positive immune responses [10,11], and may contribute to reducing vaccination stress. For instance, Lee et al. [12] investigated the effects of PE (capsicum and turmeric oleoresins) in chickens immunized with an *Eimeria* prolifin protein and orally challenged with virulent oocysts of *Eimeria tenella*, and noted that a PE mixture significantly altered immune parameters against coccidiosis. In addition, the use of Chinese herbal kombucha to suppress an FMD outbreak in situ was shown to be successful due to its antiviral properties [13]. Thus, in the recent years, herbs and herbal extracts have been used to enhance immunity, reduce the risk of disease prevalence rates, and promote the growth performance of livestock [14,15,16]. The supplementation of animal diets with an extract of capsicum and turmeric has been reported to confer protective immunity against avian necrotic enteritis in experimentally *E. maxima* and *C. perfringens* -challenged broiler chickens [17]. The antiviral effects of turmeric extracts against the FMD virus were reported by Deshpande and Chapalkar [18]. However, details of the effect of PE in mitigating the adverse effect of FMD vaccination in growing pigs is scarce. We hypothesized that the supplementation of PE consisting of capsicum and turmeric oleoresins may have a synergistic effect, in reducing the adverse responses following vaccination, and enhancing the performance and health status of growing pigs vaccinated with FMD virus.

Therefore, the objective of the present study was to evaluate the efficacy of dietary supplementation of commercial PE consisting of a mixture of oleoresins from capsicum and turmeric on growth promotion, immune response, as well as antibody titer in growing pigs inoculated with FMD vaccine.

## 2. Material and Methods

The experimental protocol (DK-1-1809) describing the management and care of animals was reviewed and approved by the Animal Care and Use Committee of Dankook University, Cheonan, South Korea.

### 2.1. Tested Product

The PE used in this study was obtained from commercial company (XTRACT^®^ Nature, Pancosma, Geneva, Switzerland). The active components of the product were 4% turmeric and 4% capsicum oleoresins, presented in a microencapsulated form.

### 2.2. Experimental Animals and Vaccination

In the present experiment, a total of 120 crossed [(Landrace × Yorkshire) × Duroc] growing pigs with an average initial BW of 24.66 ± 2.34 kg and an average age of 70 days were randomized into three groups (10 pens; 4 pigs per pen per treatment) as follows: a nonvaccinated, negative control group (NV group, n = 40), which received no PE supplementation or FMD vaccination; an only-vaccinated group (OV group, n = 40), which received the FMD vaccination but no PE supplementation; and a third group which received a PE blend (powdered form) supplement in the diet (0.125 gm/kg feed), was fed ad libitum, and received the FMD vaccination and 0.0125% of PE in a 6-week trial. After recording BW and feed consumption at week 2, FMD vaccination was carried out with 2 mL of the Aftogen Oleo FMD vaccine (Biogenesis Bago, Buenos Aires, Argentina) according to the manufacturer’s instructions for OV and PV groups. This vaccine contains inactivated FMD virus antigens (FMD 01 Campos virus inactivated BEI). One shot of vaccine was injected intramuscularly at the back of the ear on the 8th day of the trial (i.e., at 78 days of age).

### 2.3. Animal Housing and Feed

All animals were housed in an environmentally controlled growing facility with slatted plastic flooring and a mechanical ventilation system. The temperature was maintained at 25 °C for the experiment period. Each pen was allowed ad libitum access to feed and water through a self-feeder and nipple drinker throughout the experimental period. Diets were formulated to meet or exceed NRC [19] recommendations for all nutrients (Table 1).

### 2.4. Rectal Temperature

Rectal temperature was measured daily from 10 randomly selected pigs (one pig per pen) per treatment using a digital electronic thermometer once a day (14:00 h), and data are presented on a weekly basis.

### 2.5. Experimental Procedures, Sampling, and Analysis

#### 2.5.1. Animal Performance

Individual body weight was recorded initially, at week 2, and at week 6 of the experimental period. Feed consumption was recorded on a pen basis in weeks 2 and 6 of the experiment to calculate ADG, average daily feed intake (ADFI), and G:F.

#### 2.5.2. Nutrient Digestibility

An indigestible inert marker, chromium oxide (0.20%) was added to the diet for 7 days prior to fecal collection at the 6th week to calculate DM and nitrogen (N) digestibility. Fecal samples were collected randomly from at least two pigs (one barrow and one gilt) from each pen, mixed, and pooled, and a representative sample was stored in a freezer at −20 °C until it was analyzed. Before chemical analysis, the fecal samples were thawed and dried at 60 °C for 72 h, after which they were finely ground to pass through a 1 mm screen, and analyzed for N (method 968.06; [20]), Ca (method 984.01; [20]), P (method 965.17; [20]), crude fat (method 920.39 [20]), and crude fiber (method 962.09 [20]). The calcium content was determined by colorimetric assay following digestion with 6M HCl to release Ca according to (method 968.08D [21]). Feed samples were ashed and P was determined colorimetrically at 680 nm according to AOAC (method 968.08D; [21]). Lysine was measured after 24 h hydrolysis in HCl using an AA analyzer (Beckman 6300; Beckman Coulter Inc., Fullerton, CA, USA). Methionine and cysteine were determined as Met sulfone and cysteic acid after cold performic acid oxidation overnight before hydrolysis (method 982.30 E (b); [21]). Nitrogen was determined (Kjectec 2300 Nitrogen Analyzer; Foss Tecator AB, Hoeganaes, Sweden), and CP was calculated as N × 6.25. Chromium was analyzed via UV absorption spectrophotometry (Shimadzu UV-1201, Shimadzu, Kyoto, Japan) following the method described by Williams et al. [22]. The digestibility was calculated using the following formula:

Digestibility (%) = {1 − [(N_f_ × C_d_)/(N_d_ × C_f_)]} × 100 (1)

where N_f_ = nutrient concentration in feces (% DM), N_d_ = nutrient concentration in diet (% DM), C_d_ = chromium concentration in diets, C_f_ = Chromium concentration in feces.

#### 2.5.3. Blood Parameters

At 10, 15, 20, and 25 days postvaccination, ten pigs (1 pig per pen) were randomly chosen from each treatment. Blood samples were collected from the same pigs at each collection time by jugular venipuncture into vacuum tubes containing no additive (Becton Dickinson Vacutainer Systems, Franklin Lakes, NJ, USA) to obtain serum. The serum was separated by centrifugation for 15 min at 3000× *g* at 4 f after 1 h of collection, and serum samples were maintained at −4 °C until haptoglobin analyses with an automatic blood analyzer (Advia 120, Bayer, Tarrytown, NY, USA). C-reactive protein (CRP), TNF-γ, INF-γ and IL-6 were measured using commercial enzyme-linked immuno sorbent assay (ELISA) kits (IBL International GmbH, Hamburg, Germany). The presence of antibody titers against FMDV in inactivated sera from 8 randomly selected pigs (2 each from 4 randomly selected pens and pooled on a pen basis) per treatment was assayed using the Prio CHECK FMDV type O ELISA kit (Prionics AG, Zurich, Switzerland). Antibody titer against FMDV in sera was indicated by the inhibition percentage (PI), and all procedures were performed following the manufacture’s protocols.

### 2.6. Statistical Analyses

The data were analyzed using the GLM procedure of SAS (SAS Institute, Cary, NC, USA, 2002) in a randomized complete block design. The pen served as the experimental unit. Preplanned contrasts were used to test the main effects of the FMD vaccine in (i) Only vaccinated vs. Nonvaccinated groups (OV vs. NV), (ii) Vaccinated group without PE supplement vs. Vaccinated group with PE supplement (OV vs. PV), and (iii) NV vs. PV. The significance level was set at *p* < 0.05.

## 3. Results

### 3.1. Growth Performance

As shown in Table 2, the growth performance between NV versus OV and the NV vs. PV groups were comparable. However, the PV group receiving the supplementation of PE blend significantly increased (*p* < 0.05) the BW compared with the OV group at weeks 2 and 6. In addition, the ADG (at week 2 and overall) and G:F at week 2 were significantly higher (*p* < 0.05) in the PV compared with the OV group.

### 3.2. Digestibility

The apparent total tract digestibility (ATTD) of DM and nitrogen were comparable between NV and OV group (Table 3). However, the ATTD of DM at week 6 was higher (*p* < 0.05) in the PV compared with the OV group, whereas the ATTD of nitrogen was not affected between the PV and OV groups.

### 3.3. Blood Metabolites

The blood serum metabolites (haptoglobin, C-reactive protein, TNF-α, INF-γ, and IL6) concentrations measured at days 10, 15, 20, and 25 postvaccination did not differ significantly among the NV, OV, and PV groups. However, a significant increase (*p* < 0.05) in haptoglobin concentration was observed in OV group compared with NV at 25 days postvaccination (Table 4).

**Figure 1:**
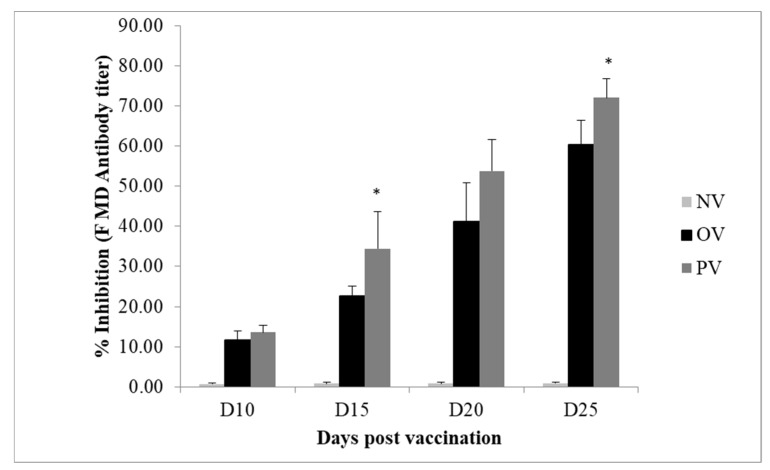
Pigs were vaccinated intramuscularly with or without 2 mL of Aftogen Oleo Foot and Mouth Disease (FMD) vaccine that contained inactivated FMD virus antigen (FMD 01 Campos virus inactivated BEI) at 78 days of age. Percentage inhibition of FMD virus in pigs without FMD vaccination (NV), with FMD vaccination (OV) and Plant extract (0.012% capsicum and turmeric oleoresins) supplemented to vaccinated pigs (PV) at days 10, 15, 20 and 25 postvaccination. * *p* < 0.05.

As shown in Figure 1, the inhibition percentage of antibodies against FMD antigens in the sera was significantly higher (*p* < 0.05) in the OV compared to the NV group at days 10, 15, 20, and 25 postvaccination. In addition, the inhibition percentage of antibodies against FMD antigens in the sera was significantly higher (*p* < 0.05) in the PV than in the OV group at d 15 and d 25 postvaccination. At day 20, a trend of higher (*p* = 0.087) inhibition percentage of antibodies against FMD antigens was observed in PV compared to the OV group.

### 3.4. Rectal Temperature

**Figure 2:**
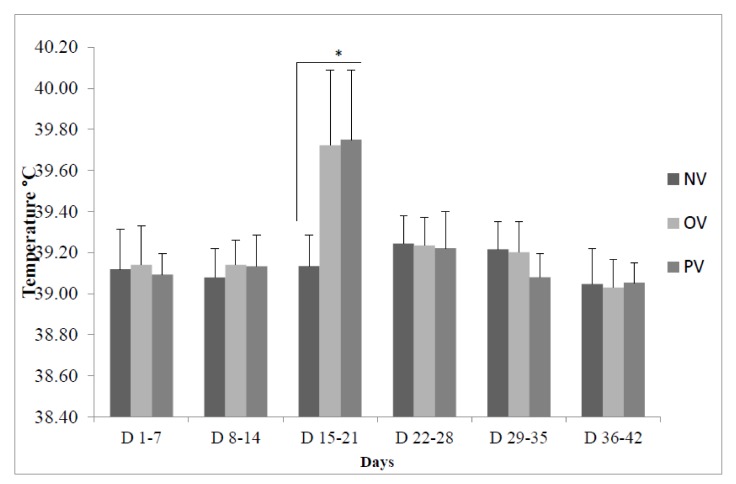
Rectal temperature of pigs (n = 10 per treatment) without vaccination (NV), with FMD vaccination (OV) and plant extract supplemented to vaccinated animal (PV) during days 1 to 42. FMD vaccine was injected intramuscularly at the back of the ear on the 8th day of the trial (78 days of age). * *p* < 0.05.

The rectal temperature did not differ significantly among the three groups at weeks 1, 2, 4, 5, and 6. However, during week 3, the rectal temperature was higher (*p* < 0.05) in the OV and PV groups compared to the NV group (Figure 2).

## 4. Discussion

### 4.1. Phytonutrient Improve Animal Performance

PE supplementation not only shows immune modulating properties, but also has a positive influence on animal performance. In a study, Jo et al. [23] demonstrated that FMD vaccination decreased the ADG of goats without affecting diet intake. In the current study, a slight reduction in ADG was observed in an FMD vaccinated pig compared with nonvaccinated animals. However, the supplementation of 0.0125% PE (turmeric and capsicum oleoresins) to FMD vaccinated pigs increased BW, ADG, and G:F at week 2, and ADG was also significantly increased overall compared with vaccinated pigs without PE supplementation, indicating the potency of PE to enhance daily gain and feed efficiency in FMD immunized animals. However, the ADFI was not affected with the supplementation of PE. Lee at al. [24] also demonstrated that the supplementation of PE containing 200 ppm capsicum oleoresin and 200 ppm of turmeric oleoresin increased ADG overall during the experimental period compared with the control group, but did not affect ADFI in weaning pigs. The improvement in growth performance in pigs receiving PE supplementation (PV) in comparison to the OV might be due to the reduction of vaccine-related stress resulting from immune stimulation after vaccination. In the current experiment, the digestibility of DM was also positively influenced by the inclusion of PE into the diet, but no effects of PE were observed for nitrogen digestibility. This better DM digestibility might be due to the reduction in pathogenic microbes and the increase in beneficial gut microbes in the intestine induced by the capsicum oleoresin. The plant extracts tested reduced diarrhea and inflammation caused by *E. coli* infection, which may be beneficial to pig health [25]. However, Maneewan et al. [26] demonstrated that the inclusion of graded level of turmeric improved the digestibility of crude fat, crude protein, and crude fiber, but had no effect on DM and energy digestibility in weaning pigs. The inconsistent results might be due to the inclusion level of the PE and the age of the pigs.

### 4.2. Phytonutrient Did Not Affect Immune Response Measured after 10 Days of Vaccination

Vaccination is considered to be one of the greatest medical achievements in reducing the mortality rate related to various infectious diseases [27,28]. Mild local and systemic reactions to vaccines are likely to be a natural consequence of the vigorous stimulation of the immune system. Recently, various medicinal plants have been used as an adjuvant for vaccine antigens pertaining to the enhancement of antibodies and cell-mediated immune response [8,29]. The present study evaluated the application of PE as a feed additive in FMD-vaccinated animals to test its effects on immune markers such as TNF-α, INF-γ, and IL-6, acute phase proteins such as haptoglobin, and C-reactive proteins. The concentrations of these immune related indices were not significantly different among the three groups after 10 days of vaccination, except for the haptoglobin levels, that were higher in the OV than the NV group at 25 days postvaccination. A previous study also demonstrated no IFN-γ activity in FMDV-vaccinated pigs [30]. The rectal temperature for the OV and PV groups were higher during week 3. The reasons for the increase in haptoglobin at day 25 postvaccination and rectal temperature during week 3 for the OV and PV groups are unclear. The lack of effects on immune-related markers in the OV and PV groups compared with the NV group could be related to the time selected for the measurement of immune markers postvaccination. Probably, an intensive schedule of sample collection may help to detail the stimulation of immune response following vaccination. We may have observed a difference between treatments if sampling had been done during the first 2–3 days postvaccination, or it could be due to the single vaccination dose.

Antibody levels are known correlates of vaccine-induced immunity. In the current study, the inhibition percentage of antibodies against FMD antigens was higher in the OV group than in the NV group. In addition, the pigs in PV group exhibited significantly higher PI values against FMD antigens compared with OV group, which could be from better vaccine related immune response due to reduced vaccine stress in the PE supplemented group. Sera with a PI value greater than 50% were considered seropositive in the ELISA assays. A study by Fu et al. [13] also demonstrated the effectiveness of a Chinese herbal extract made from licorice, luhanguo, chrysthemum, and Chinese tea in the inhibition of the FMD virus in BHK21 cells and suckling mice. The simultaneous administration of PE consisting of carvacrol, cinnamaldehyde, and capsicum oleoresin with the Newcastle disease vaccine enhanced immunoglobulin production in broiler chickens, indicating that PE could partially counter immune dysfunction [31]. In addition, the antiviral activity of PE obtained from turmeric, Ashwangandha, and Tulsi plant against FMDV has been reported; but the mechanism for this was unclear if it was due to the inhibition of phytochemicals for viral attachment, entry, replication assembly, and transmission from one cell to other [18]. However, to date, the antiviral mechanisms of action for PE have not been adequately evaluated. Thus, further studies pertaining to the observed mechanism are recommended.

## 5. Conclusions

The findings of this study suggest that PE supplementation to FMD-vaccinated growing pigs alleviates the negative effect of vaccination on body weight, and showed a significant positive effect on ADG, G:F, and an increase in DM digestibility in vaccinated animals. Furthermore, it conferred antiviral effects by increasing the antibody titer compared to that in animals without PE supplementation. The cytokines and acute phase protein levels were unaltered among the treatment groups, although haptoglobin concentrations were higher in the OV group 25 days postvaccination compared to the NV group; the reason for this is unclear.

## Figures and Tables

**Table 1:** Composition of basal diet (as fed-basis, g/kg).

Items	
Corn	375.7
Wheat	190
Rice bran	20
Wheat bran	20
Palm kernel meal	20
Soybean meal	30
De-hulled soybean meal	151.1
Rape seed meal	40
Sesame meal	20
Brown Rice	50
Animal fat	37.9
Molasses	20
Limestone	10.5
MCP	1.6
Salt	3.0
Methionine 98%	0.1
Threonine 98%	0.2
Lysine 25%	5.0
Choline Chloride 50%	0.9
Vitamin/Mineral mixture *	4.0
Calculated composition	
Digestible Energy (MJ/kg)	14.90
Analyzed composition, %	
Crude Protein	17.50
Crude Fat	6.7
Crude Ash	4.4
Crude Fiber	3.8
Total Lysine	0.99
Calcium	0.75
Phosphorus	0.42

* Provided per kg of complete diet: 11,025 IU vitamin A; 1103 IU vitamin D3; 44 IU vitamin E; 4.4 mg vitamin K; 8.3 mg riboflavin; 50 mg niacin; 4 mg thiamine; 29 mg D-pantothenic; 166 mg choline; 33 μg vitamin B12. Provided per kg of complete diet: 12 mg Cu (as CuSO_4_·5H_2_O); 85 mg Zn (as ZnSO_4_); 8 mg Mn (as MnO_2_); 0.28 mg I (as KI); 0.15 mg Se (as Na_2_SeO_3_·5H_2_O).

**Table 2:** Effect of plant extract supplementation on growth performance in FMD vaccinated growing pigs *.

Items	NV	OV	PV	SEM ^†^	*p*-Value		
					OV vs. NV	OV vs. PV	NV vs. PV
Body weight, kg							
Initial	24.66	24.65	24.68	0.006	0.5542	0.0700	0.2014
wk2	33.11	32.98	33.36	0.11	0.4074	0.022	0.1147
wk6	53.89	53.46	54.51	0.31	0.3397	0.0291	0.1816
Week 2							
ADG, g	603	594	621	8.0	0.4305	0.0298	0.1377
ADFI, g	1228	1229	1249	11.0	0.9735	0.2131	0.2027
G/F	0.491	0.484	0.496	0.004	0.1393	0.0749	0.735
Week 6							
ADG, g	742	731	755	10.1	0.4565	0.1	0.3724
ADFI, g	1652	1650	1655	13.2	0.9307	0.8021	0.8699
G/F	0.449	0.444	0.456	0.008	0.5752	0.2686	0.5752
Overall							
ADG, g	696	686	710	8.1	0.348	0.0318	0.1896
ADFI, g	1511	1510	1520	11.2	0.9529	0.5278	0.5664
G/F	0.461	0.455	0.467	0.006	0.4795	0.1659	0.4795

* Abbreviation: NV, Non FMD vaccinated group; OV, FMD vaccinated group only; PV, plant extract blend (0.0125%) supplemented to FMD vaccinated group; ^†^ Standard error of means.

**Table 3:** Effect of plant extract supplementation on coefficient of apparent total tract nutrient digestibility in FMD vaccinated growing pigs *.

Items	NV	OV	PV	SEM ^†^	*p*-Value		
					OV vs. NV	OV vs. PV	NV vs. PV
Week 6							
Dry matter	0.758	0.753	0.770	0.0044	0.4809	0.0213	0.0828
Nitrogen	0.744	0.743	0.755	0.0052	0.9425	0.1234	0.1397

* Abbreviation: NV, Non FMD vaccinated group; OV, FMD vaccinated group only; PV, plant extract blend (0.0125%) supplemented to FMD vaccinated group; ^†^ Standard error of means.

**Table 4:** Effect of plant extract supplementation on blood profile in FMD vaccinated growing pigs *.

Items	NV	OV	PV	SEM ^†^	*p*-Value		
					OV vs. NV	OV vs. PV	NV vs. PV
Postvaccination Day 10							
Haptoglobin, mg/dL	15.8	17.3	16.5	1.3	0.4286	0.6709	0.7099
C-Reactive Protein, mg/L	10.8	13.5	12.3	2.7	0.4895	0.7594	0.6978
TNF- α, U/mL	14.4	18.9	16	2.4	0.1937	0.3957	0.6371
INF-γ ng/mL	33.5	38.7	35.9	2.7	0.1961	0.479	0.5432
IL-6, pg/mL	15.4	19.7	18.4	4.0	0.3854	0.791	0.5426
Postvaccination Day 15							
Haptoglobin, mg/dL	17.2	19.2	18.7	1.2	0.2607	0.7749	0.3953
C-Reactive Protein, mg/L	12	15.3	14.3	1.9	0.2431	0.7268	0.4055
TNFx α, U/mL	16.3	20.3	18.9	2.5	0.2693	0.6946	0.4683
INF- γ, ng/mL	33	38.5	35	3.1	0.2258	0.4352	0.6538
IL-6, pg/mL	18.7	22.6	20.9	5.8	0.6335	0.8349	0.7875
Postvaccination Day 20							
Haptoglobin, mg/dL	15.9	17.5	16.2	1.2	0.3525	0.4481	0.860
C-Reactive Protein, mg/L	11.7	13.4	12.6	1.7	0.4843	0.7622	0.6889
TNF- α, U/mL	15.2	18.4	17.2	2.3	0.3365	0.7155	0.5448
INF- γ, ng/mL	31.9	36.9	34.6	3.7	0.3548	0.6674	0.6143
IL-6, pg/mL	14.4	18.7	17.5	3.6	0.4118	0.8172	0.5522
Postvaccination Day 25							
Haptoglobin, mg/dL	13.6	15.3	14.6	0.6	0.0485	0.395	0.2291
C-Reactive Protein, mg/L	9.6	12.4	10	1.3	0.1369	0.202	0.8186
TNF-α, U/mL	12.1	15.9	14.4	2.1	0.2065	0.6112	0.438
INF- γ, ng/mL	29.8	31.4	30.4	3.8	0.7688	0.8541	0.9121
IL-6, pg/mL	12.3	14.0	13.1	2.1	0.5741	0.7653	0.7907

* Abbreviation: NV, Non FMD vaccinated group; OV, FMD vaccinated group only; PV, plant extract blend (0.0125%) supplemented to FMD vaccinated group; ^†^ Standard error of means.3.4. Antibody Titer.
